# EphA4 Obstructs Spinal Cord Neuron Regeneration by Promoting Excessive Activation of Astrocytes

**DOI:** 10.1007/s10571-021-01046-x

**Published:** 2021-02-17

**Authors:** Xiaogang Chen, Lin Zhang, Fu Hua, Yu Zhuang, Huan Liu, Shouguo Wang

**Affiliations:** 1 Department of Orthopaedics, The Affiliated Huaian No. 1 People’s Hospital of Nanjing Medical University, No 6, Beijing West Road, Huai’an, 223300 Jiangsu People’s Republic of China; 2 Department of Gynecology, The Affiliated Huaian No. 1 People’s Hospital of Nanjing Medical University, Huai’an, Jiangsu People’s Republic of China

**Keywords:** EphA4, Spinal cord injury, Neurite outgrowth and regeneration, Astrocyte function, Ephrin-B

## Abstract

Studies have found that molecular targets that regulate tissue development are also involved in regulating tissue regeneration. Erythropoietin-producing hepatocyte A4 (EphA4) not only plays a guiding role in neurite outgrowth during the development of the central nervous system (CNS) but also induces injured axon retraction and inhibits axon regeneration after spinal cord injury (SCI). EphA4 targets several ephrin ligands (including ephrin-A and ephrin-B) and is involved in cortical cell migration, axon guidance, synapse formation and astrocyte function. However, how EphA4 affects axon regeneration after SCI remains unclear. This study focuses on the effect and mechanism of EphA4-regulated astrocyte function in neuronal regeneration after SCI. Our research found that EphA4 expression increased significantly after SCI and peaked at 3 days post-injury; accordingly, we identified the cellular localization of EphA4 and ephrin-B ligands in neurons and astrocytes after SCI. EphA4 was mainly expressed on the surface of neurons, ephrin-B1 and ephrin-B3 were mainly localized on astrocytes, and ephrin-B2 was distributed on both neurons and astrocytes. To further elucidate the effect of EphA4 on astrocyte function after SCI, we detected the related cytokines secreted by astrocytes in vivo. We found that the levels of neurotrophic factors including nerve growth factor (NGF) and basic fibroblast growth factor (bFGF) increased significantly after SCI (NGF peaked at 3 days and bFGF peaked at 7 days); the expression of laminin and fibronectin increased gradually after SCI; the expression of inflammatory factors [interleukin (IL)-1β and IL-6] increased significantly from 4 h to 7 days after SCI; and the levels of glial fibrillary acidic protein (GFAP), a marker of astrocyte activation, and chondroitin sulphate proteoglycan (CSPG), the main component of glial scars, both peaked at 7 days after SCI. Using a damaged astrocyte model in vitro, we similarly found that the levels of related cytokines increased after injury. Consequently, we observed the effect of damaged astrocytes on neurite outgrowth and regeneration, and the results showed that damaged astrocytes hindered neurite outgrowth and regeneration; however, the inhibitory effect of injured astrocytes on neurite regeneration was reduced following ephrin-B receptor knockdown or inflammatory inhibition at 24 h after astrocyte injury. Our results showed that EphA4 regulates the secretion of neurotrophic factors, adhesion molecules, inflammatory factors and glial scar formation by binding with the ligand ephrin-B located on the surface of astrocytes. EphA4 affects neurite outgrowth and regeneration after SCI by regulating astrocyte function.

## Introduction

After spinal cord injury (SCI), Eph family members and their ligands (ephrins) accumulate in the proximal axon stump and reactive astrocytes and play an important role in the formation of glial scars and neurite regeneration (Bundesen et al. 2003). Clustered EphA4 reduces the expression of glutamate transporters and glutamate absorption of astrocytes by activating the reverse signal transduction of ephrin-A3, indicating that EphA4-mediated ephrin-A3 reverse signal transduction is an important mechanism by which astrocytes control glial glutamate transporters and prevent glutamate excitotoxicity under pathological conditions (Wegmeyer et al. 2007). Some research found that the number of astrocytes and the area of glial scars were significantly reduced after lateral spinal cord hemisection in *epha4*^−/−^ mice (Herrmann et al. 2010); in contrast, *epha4* gene deletion did not significantly affect the reactive proliferation of astrocytes or promote the formation of astrocyte fibrosis scar after spinal cord hemisection in mice (Goldshmit et al. 2004). This discrepancy cannot be explained by different animal models, Turnley et al*.* re-examined the proliferation of astrocytes after injury in *epha4* null mice and found that the reactivity of astrocytes in the injured area did not decrease, and the degree of other phenotypes such as that of the spinal dorsal cord did not change. These findings also demonstrated that the EphA4-mediated astrocyte phenotype changes were related to the aggregation of EphA4 but not to the strain background of the mice. The results suggest that EphA4 expression in the injured area after SCI is involved in the regulation of astrocyte function, which also affects the regeneration and functional reconstruction of neuronal processes after SCI.

The signal transduction pathway that regulates tissue formation during development is also involved in the regulation of tissue regeneration; consequently, elucidation of the regulation of CNS development may suggest important strategies for nerve repair after injury (Parmentier-Batteur et al. 2011). The Eph family of receptor tyrosine protein kinases not only guides neurite outgrowth during development but also regulates axon guidance and dendrite function after SCI. At present, 16 Eph receptors and 9 ephrin ligands have been found. According to sequence homology, Eph receptors can be divided into EphA (epha1–epha10) and EphB (ephb1–ephb6); according to their structures, ephrin ligands can be divided into two subtypes: the ephrin-A subtype (ephrin-a1–ephrin-a6), which is immobilized on the cell membrane by glycosylphosphatidylinositol (GPI), and ephrin-B, which belongs to the transmembrane protein family subtype (ephrin-b1–ephrin-b3). Although ephrin ligands bind to Eph receptors at will, they still have some selectivity; generally, ephrin-A ligands preferentially bind to EphA receptors, while ephrin-B ligands preferentially bind to EphB receptors. One important feature of the ephrin family is that it has a two-way signal transduction function: the classical tyrosine kinase pathway via the Eph receptor and the reverse signal transduction pathway via ephrin ligands (Aoto and Chen 2007; Klein 2009). EphA4 can bind to all ephrin-A and ephrin-B ligands, and the combination of EphA4 and ephrin-B2 precisely regulates the migration of cortical neurons during development (Todd et al. 2017). The EphA4/ephrin-A3 signalling pathway is the key pathway by which astrocytes regulate synaptic function and plasticity (Filosa et al. 2009; Yang et al. 2014).

Most of the results suggest that inhibition of EphA4 expression is not conducive to the overactivation and proliferation of astrocytes but is conducive to the regeneration of neurites, which promotes control of the local inflammatory response. Therefore, inhibition of EphA4 can provide a favourable environment for functional repair after SCI. However, moderate activation and proliferation of glial cells and inflammatory response are beneficial to repair and limit the extent of injury because EphA4 binds with many ligands to perform different functions, and the interaction between ephrin ligands and Eph receptors has a two-way conduction mechanism. It is very important to choose an appropriate time point to intervene in the expression of EphA4 and clarify the function of EphA4 binding with different ligands to promote functional recovery after SCI. We focused on the role of EphA4 in regulating the function of astrocytes through binding to ephrin-B ligands after SCI and the effects of damaged astrocytes on neurite outgrowth and regeneration. Our results indicate that EphA4 binding to different ephrin-B ligands simultaneously affects the outgrowth and regeneration of neuronal processes after SCI.

## Materials and Methods

### Mouse Model of SCI

The animal experiments were approved by the ethics review committee of the affiliated Huaian No. 1 People’s Hospital of Nanjing Medical University, China. A total of 105 C57BL/6 mice (5 months old, male) and 21 *epha4*^−/−^ mice (5 months old, male) were purchased from Beijing Charles River Experimental Animal Center, China. The animals were housed in a ventilated room at a constant temperature (24 °C), 50–60% humidity, and a 12-h light/dark cycle with a standard mouse diet and free access to water. After 2 weeks of feeding, the spinal cord was subjected to dorsal hemisection at the T9 segment of the spinal cord. The operation was performed on a constant temperature operating table at 37 °C. The mice that underwent laminectomy were used as the sham group. After successful modelling, the bladder was emptied manually every 4 h. All C57BL/6 mice were randomly divided into 7 groups: the sham group (*n* = 15) and the SCI group (including different time points: 4 h, 1 day, 3 days, 7 days, 14 days and 28 days) (*n* = 90). In each group, 5 animals were used for immunofluorescence staining, 5 for mRNA detection and 5 for protein detection. The *epha4*^−/−^ mice were also divided into 7 groups, and three experimental animals in each group were used to detect cytokines and astrocyte activity.

### Real-Time PCR Assay

One centimetre of spinal cord tissue containing the central area of SCI was taken. After addition of 800 μL of TRIzol (Invitrogen, USA) to the tissue, the tissue was homogenized, 200 μL of chloroform (Sinopharm Group Co., Ltd., China) was added to the tissue, and the sample was shaken violently for 15 s and then kept at 4 °C for 15 min. The tissue was centrifuged at 4 °C and 12,000 rpm for 15 min, and the centrifuged upper transparent aqueous phase was transferred into a new RNase-free centrifuge tube. Then, 500 μL of isopropanol was added to the tube, and the tube was kept at 4 °C for 15 min and centrifuged at 12,000 rpm for 15 min. The supernatant was discarded, and the RNA pellet was washed with 75% ethanol [absolute ethanol with diethyl pyrocarbonate (DEPC) water] (Sinopharm Group Co., Ltd., China) and dried. After the RNA was dried, an appropriate amount of DEPC water was added, and the RNA concentration and purity were determined by ultraviolet spectrophotometry and stored at − 80 °C.

Quantitative PCR detection of genes was performed using SYBR® Green Real-time PCR Master Mix (Toyobo, Japan). A 20-μL reaction system was used, with the following three-step PCR cycle: predenaturation at 95 °C for 60 s, followed by 40 cycles of 95 °C for 15 s, 60 °C for 15 s and 72 °C for 45 s. Melting curve analysis was performed, and the relative amount of the detected gene were calculated according to the formula 2^−ΔΔCT.^ glyceraldehyde-3-phosphate dehydrogenase (GAPDH) was used as the internal reference. The following primer sequences were used: EphA4—forward: 5′-TGGAATTTGCGACGCTGTCA-3′; reverse: 5′-CACTTCCTCCCACCCTCCTT-3′, and GAPDH—forward: 5′-CTTGCTCAAGCTTAGTTCTAGG-3′; reverse: 5′-GAGTGCTCAGTGGTATTGC -3′.

### Western Blot Assay

One centimetre of spinal cord tissue containing the central area of SCI was taken for protein extraction. Briefly, spinal cord tissue was homogenized in 1 mL of RIPA solution (Beyotime, China) and centrifuged at 12,000 rpm for 15 min at 4 °C, and the supernatant was collected for protein quantification. Twenty micrograms of protein was loaded onto a sodium dodecyl sulphate–polyacrylamide gel electrophoresis (SDS-PAGE) gel and transferred to a PVDF membrane (Millipore, USA). After the membrane was blocked at room temperature for 30 min, EphA4, ephrin-B1, ephrin-B2 and ephrin-B3 primary antibodies (Abcam, USA) were separately incubated overnight at 4 °C, goat anti-mouse HRP (CST, USA) or goat anti-rabbit HRP (CST, USA) secondary antibodies were used in combination with primary antibodies, and an ECL Plus kit was used to detect the positive bands with an Odyssey dual colour infrared laser imaging system (Odyssey, USA). GAPDH was used as the internal reference. Protein quantification was performed using IPP software, and the differences between groups were determined.

### Immunofluorescence Staining

Each mouse was anaesthetized with 200 μL of 2% sodium pentobarbital (Sinopharm Group Co., Ltd., China), perfused with 50 mL of normal saline (Sinopharm Group Co., Ltd., China), and fixed with 50 mL of precooled 4% paraformaldehyde (Sangon, China). The perfused mice were placed at room temperature for 4 h, and spinal cord tissues were collected and placed in paraformaldehyde at 4 °C overnight following complete fixation. After dehydration with 20% and 30% sucrose (Sinopharm Group Co., Ltd., China), the spinal cord tissues were frozen (15 μm/section) and cryopreserved at − 80 °C. For immunofluorescence staining, the tissue sections were taken from the refrigerator and placed at room temperature for 5 min, washed with PBS for 5 min and then blocked with blocking solution containing 5% normal goat serum at room temperature for 30 min. For localization of EphA4, ephrin-B1, ephrin-B2 and ephrin-B3 in neurons and astrocytes, we mixed rabbit EphA4 (1:500, Abcam, USA), ephrin-B1 (1:500, Abcam, USA), ephrin-B2 (1:500, Abcam, USA) and ephrin-B3 (1:500, Abcam, USA) primary antibodies with mouse β-tubulin (1:100, CST, USA) and GFAP (1:500, Boster, China) monoclonal antibodies in one primary antibody dilution, and the dilution was incubated with the sections overnight at 4 °C. The sections were washed with 0.01 M PBS for 10 min, 3 times. Goat anti-mouse 488 secondary antibody and goat anti-rabbit 555 secondary antibody (Beyotime, China) were diluted 1:300 in secondary antibody dilution and incubated for 2 h at room temperature before washing with 0.01 M PBS for 10 min, 3 times. DAPI (Beyotime, China) staining solution was added dropwise to the tissue sections, and the sections were covered with coverslips. Fluorescent staining pictures were observed and captured under an upright fluorescence microscope (Olympus, Japan).

### Primary Neuron and Spinal Cord Slice Culture or Coculture with Astrocytes In Vitro

Mouse spinal cord tissue from 14-day-old embryos was collected for primary spinal cord neuron culture. The tissue was cut into pieces by sterile ophthalmic scissors and digested with 0.05% trypsin-ethylenediamine tetraacetic acid (EDTA) (Gibco, USA) at 37 °C for 15 min. A pipette was used to blow the tissue into a single cell suspension. Dulbecco's modified Eagle's medium (DMEM) + 10% foetal bovine serum (FBS) (Gibco, USA) was used as the basic culture medium for 4 h. When the cells adhered to the dishes, the culture medium was replaced by neurobasal + 1% N2 + 2% B27 (Thermo Scientific, USA) for 3 days to prevent the excessive growth of astrocytes.

Spinal cord tissue from C57BL/6 embryonic (E) 14-day-old mice was collected for the spinal cord slice culture. The whole spinal cord was placed in precooled sterile PBS and cut into small slices with sterile eye scissors. A sterile coverslip was placed in each well of the 24-well plate, and 100 μL of DF12 + 10% FBS culture medium was added to each well of the 24-well culture plate. Spinal cord slices were gently placed in the centre of the coverslip, and the plate was placed in an incubator to culture for 4 h. Subsequently, 400 μL of medium (DF12 + 10% FBS) was gently added to avoid floating spinal cord slices, and the slices were cultured for another 5 days. For the injury model, the neurites of the spinal cord slices were cut with a sterile surgical blade, and the slices were cultured for 3 days in vitro to observe regeneration.

Mouse spinal cord tissues from 14-day-old embryos were also collected for primary spinal cord astrocyte culture. The tissue was cut into pieces by sterile ophthalmic scissors and digested with 0.05% trypsin- EDTA (Gibco, USA) at 37 °C for 15 min. A pipette was used to blow the tissue into a single cell suspension. DMEM + 10% FBS (Gibco, USA) was used as the basic culture medium, and the medium was changed every other day. After 7 days of culture in vitro, the astrocyte monolayer was scraped using a 20-μL pipette tip for the astrocyte damage model. Normal astrocytes and damaged astrocytes were cocultured with spinal cord neurons or spinal cord slices to observe the effects of the damaged astrocytes on neurite outgrowth and regeneration. In the coculture system, astrocytes were directly seeded on the plate, and neurons or spinal cord slices were seeded on the coverslip in the same plate.

### Enzyme-Linked Immunosorbent Assay (ELISA)

The supernatant of spinal cord homogenate or cell culture medium was collected to detect the changes in cytokines before and after injury. In this study, Abcam's ELISA test kit was used to detect NGF, bFGF, laminin, fibronectin, IL-1β, IL-6, GFAP and CSPG levels. A microplate reader (Molecular Devices, USA) was used to read the OD value at 450 nm, and the sample concentration was calculated based on the OD value of the standard and the sample.

### Lentivirus Transfection and RNA Interference

Lentiviruses for the interference of ephrin-B1, ephrin-B2 and ephrin-B3 were all purchased from Santa Cruz (USA) with a titre of 1 × 10^8^ TU/mL. The basic medium was used to prepare a suspension of primary astrocytes at a density of 5 × 10^6^ cells/mL, and the cells were seeded in 6-cm petri dishes. When the inoculation density reached more than 90%, 400 μL of the virus infection enhancement solution HitransG A (GeneChem, China) and 100 μL of lentivirus or negative control (neg-control), each with a titre of 1 × 10^8^ TU/mL, were added, and the cells were incubated at 37 °C for 24 h. After 72 h of virus infection, the cells were collected and identified by Western blot analysis to determine whether the target protein was knocked down.

### Statistical Analysis

All statistical results were analysed using SPSS 17.0 (SPSS, Inc., Chicago, USA). All quantitative indicators are expressed as the mean ± SD. The experimental data were analysed using one-way analysis of variance and Tukey's post hoc test. *P* < 0.05 indicated significant differences between groups.

## Results

### Expression and Cellular Localization of EphA4 and ephrin-B Ligands After SCI

**Fig. 1:**
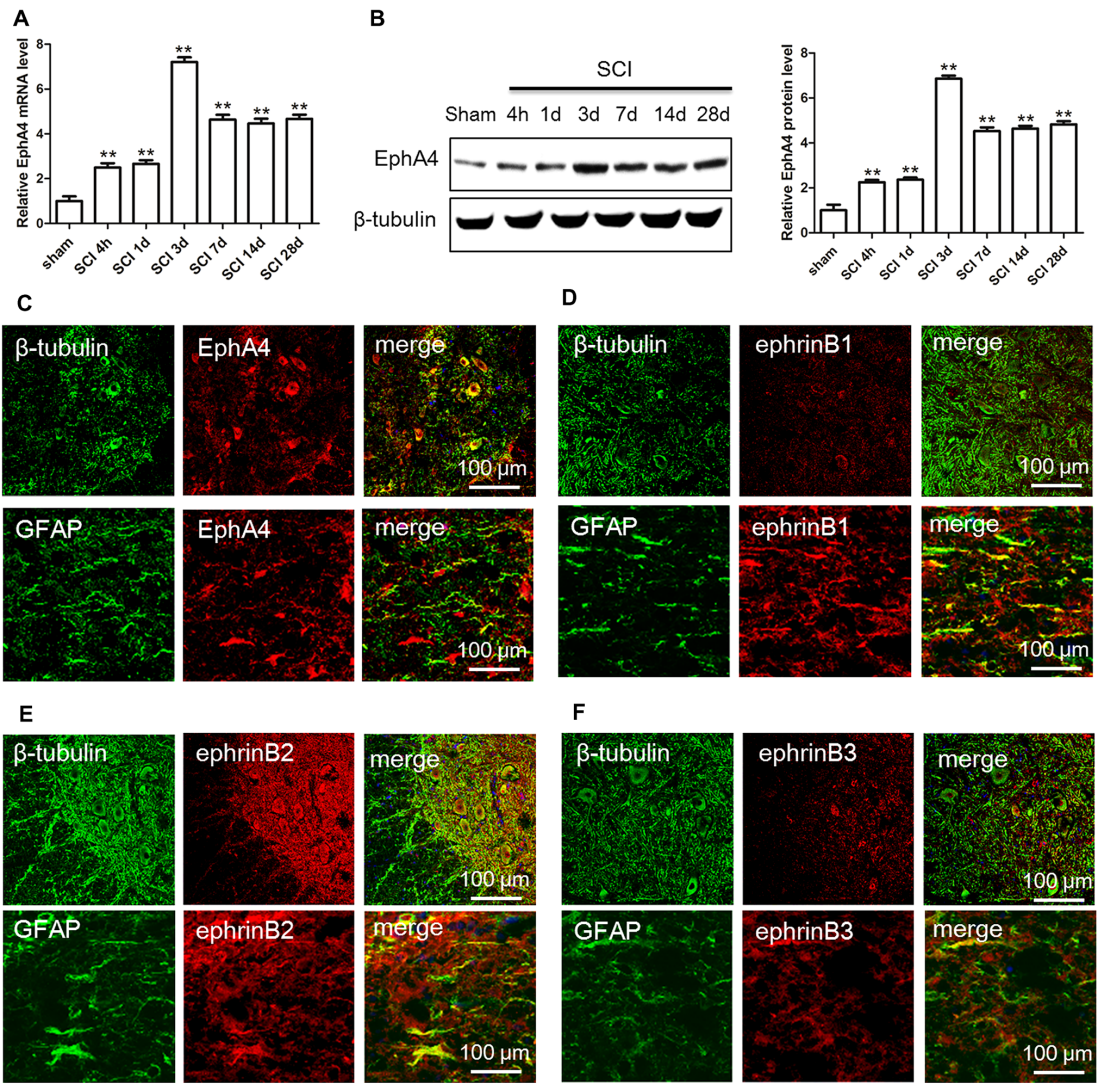
Expression and cellular localization of EphA4 and its ligands before and after SCI. **a** Real-time PCR detected the expression of EphA4 at the mRNA level after SCI. **b** Western blot analysis detected the expression of EphA4 at the protein level after SCI. **c**–**f** Immunofluorescence staining examined the localization of EphA4 and ephrin-B ligands in spinal cord neurons and astrocytes at 3 days post-injury, and the results showed that EphA4 was mainly expressed on the surface of neurons with a small amount colocalized with astrocytes; ephrin-B1 and ephrin-B3 were both barely expressed on the surface of spinal cord neurons and were mainly located in astrocytes; ephrin-B2 was widely distributed in spinal cord neurons and astrocytes. *N* = 5; **P < 0.01. Scale bar: 100 μm

To determine the effect of EphA4 binding to ephrin-B ligands on neurite outgrowth and regeneration after SCI, we first detected the expression of EphA4 and examined the cell localization of EphA4 and ephrin-B ligands after SCI. The results showed that EphA4 expression increased significantly, peaked at 3 days, and maintained at a high level from 7 to 28 days after SCI (Fig. 1a and b). Then, we detected the localization of EphA4 and ephrin-B in spinal cord neurons and astrocytes at 3 days post-injury to determine whether EphA4 regulates astrocyte function by binding to different ephrin-B ligands. Immunofluorescence double labelling showed that EphA4 was mainly expressed on the surface of neurons, and a small amount was colocalized in astrocytes (Fig. 1c); ephrin-B1 was barely expressed on the surface of spinal cord neurons and was mainly located in astrocytes (Fig. 1d); ephrin-B2 was widely distributed in spinal cord neurons and astrocytes (Fig. 1e); ephrin-B3 was distributed only on the surface of astrocytes, and no expression of ephrin-B3 was detected on the surface of neurons (Fig. 1f). Consequently, EphA4 is mainly expressed on the surface of neurons after SCI, and ephrin-B ligands are mainly expressed on the surface of astrocytes. We speculate that EphA4 participates in the regulation of astrocyte function by binding with members of the ephrin-B ligand family.

### Effects of Damaged Astrocytes on Neurite Outgrowth and Regeneration of Spinal Cord Neurons

**Fig. 2:**
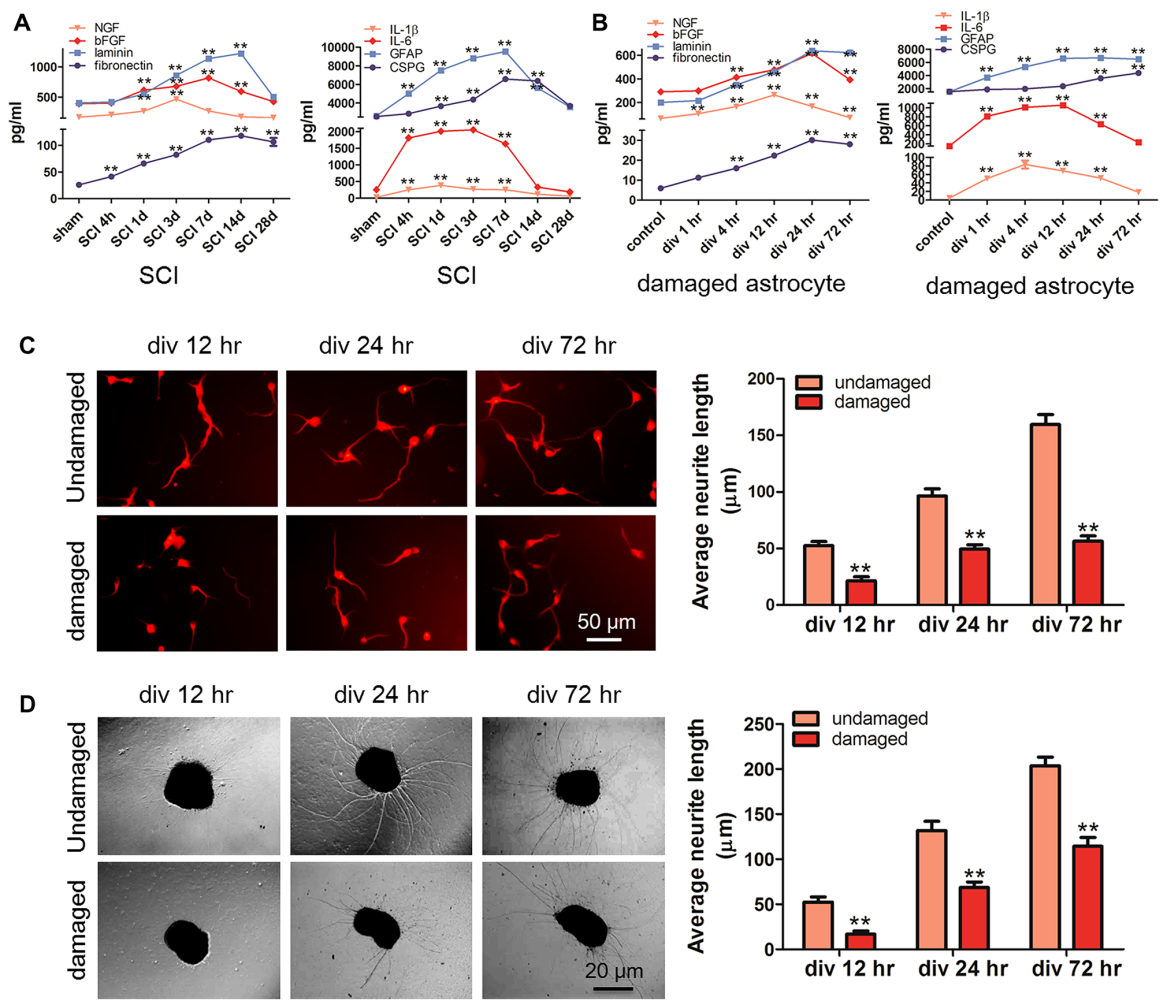
Effects of damaged astrocytes on neurite outgrowth and regeneration of spinal cord neurons. **a** ELISAs detected the expression of factors related to astrocyte function in spinal cord tissue after SCI, including NGF, bFGF, laminin, fibronectin, the inflammatory factors IL-1β and IL-6, the astrocyte marker protein GFAP and CSPG, the main component of glial scar. **b** ELISAs detected the changes in cytokines in the astrocyte injury model in vitro. **c** A neuron and astrocyte coculture system was used to examine the role of damaged astrocytes in neurons, and the results showed that damaged astrocytes hinder neurite outgrowth. **d** A spinal cord slice and astrocyte coculture system was used to examine the role of damaged astrocytes in neural regeneration, and the results showed that damaged astrocytes impeded neurite regeneration. *N* = 5; **P < 0.01. Scale bar: 20 or 50 μm

To determine the effect of astrocyte function on neurite outgrowth and regeneration after SCI, we examined factors related to astrocyte function, including NGF, bFGF, laminin, fibronectin, the cytokines IL-1β and IL-6, the astrocyte marker protein GFAP and CSPG, the main component of glial scars. ELISAs showed that the expression of NGF increased significantly only at 3 days post-injury; bFGF expression increased gradually from 1 day post-injury, peaked at 7 days and returned to the normal level at 28 days after SCI; the trend of change in laminin expression was consistent with that of bFGF; the expression of fibronectin increased gradually from 4 h to 28 days post-SCI; the expression of IL-1β and IL-6 increased significantly at 4 h post-injury and decreased significantly at 7 days post-injury; and the level of GFAP increased significantly at 4 h after injury, peaked at 7 days after injury, decreased significantly and then returned to normal at 28 days post-SCI (Fig. 2a). According to the results, we speculate that the time window for astrocytes to continuously secrete neurotrophic factors is short, while the time for secreting inflammatory factors and glial scars is longer. An environment conducive to neurite outgrowth and regeneration through regulation of the function of astrocytes is very important for the functional recovery of SCI. To further study the role of EphA4 and ephrin-B in the regulation of astrocytes, we established an astrocyte injury model in vitro and detected the changes in the above factors in the injury model over time. The results showed that the duration of high expression of neurotrophic factor in the in vitro astrocyte injury model was significantly prolonged compared with that of the in vivo injury model, which might be related to astrocyte apoptosis in the later stage of the animal model. The trends of expression of adhesion molecules, cytokines and GFAP were similar to those in vivo, the expression of CSPG was significantly increased at 24 h and 72 h after astrocyte injury, and time of high expression was significantly delayed compared with that in vivo. We speculated that the low expression of CSPG was related to the small number of astrocytes arranged in monolayers cultured in vitro (Fig. 2b). Then, we examined the effects of damaged astrocytes on neurite outgrowth and regeneration, and the results showed that compared with undamaged astrocytes, damaged astrocytes hindered the outgrowth and regeneration of neurites (Fig. 2c and d). We speculated that although the level of neurotrophic factors increased after injury, many factors hindered the outgrowth and regeneration of neurites; therefore, EphA4 regulation of the function of astrocytes needs to be further studied.

### Effects of EphA4 and Ephrin-B Ligands on Cytokine Secretion by Damaged Astrocytes

**Fig. 3:**
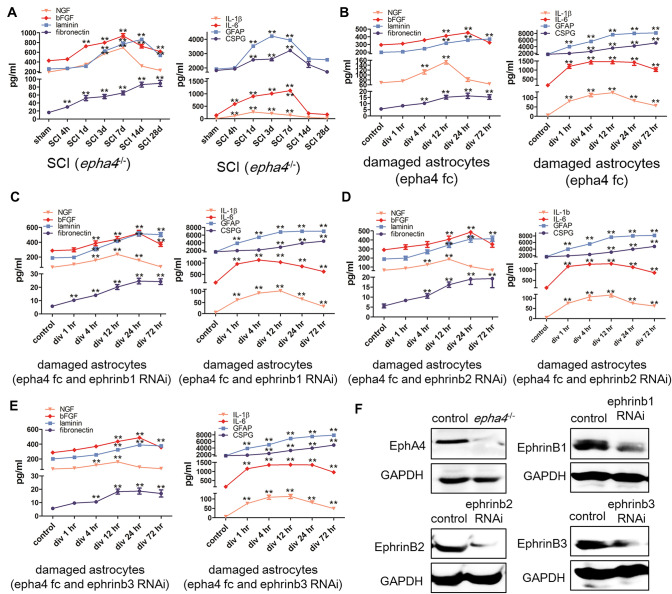
Effects of EphA4 and ephrin-B ligands on cytokine secretion by damaged astrocytes. **a** ELISAs detected the expression of cytokines related to astrocyte function in the spinal cord of the *epha4*^−/−^ mice. **b** ELISAs examined the influence of EphA4 on the expression of cytokines related to astrocyte function in damaged astrocytes. **c** ELISAs examined the influence of EphA4 on cytokine secretion by damaged astrocytes with ephrin-B1 deletion. **d** ELISAs examined the influence of EphA4 on cytokine secretion by damaged astrocytes with ephrin-B2 deletion. **e** ELISAs examined the influence of EphA4 on cytokine secretion by damaged astrocytes with ephrin-B3 deletion. **f** Western blots confirmed the deletion of EphA4 and ephrin-B ligands in cultured astrocytes. *N* = 5; **P < 0.01

To determine whether EphA4 influences the function of astrocytes in the spinal cord, we detected the expression of cytokines related to astrocyte function in the spinal cord of the *epha4*^−/−^ mice and observed whether the deletion of EphA4 affected the function of astrocytes. The levels of NGF and bFGF in the *epha4*^−/−^ mice were significantly higher than those in the normal mice, and the level of NGF increased significantly at 3 days post-injury, which was delayed compared with that of the control mice; the duration of high expression of bFGF was prolonged, and the level of bFGF was still significantly higher than that of sham group at 28 days after SCI. Compared with those of the normal mice, the levels of laminin and fibronectin in the *epha4*^−/−^ mice were significantly increased, and compared with that of the control group, the expression of laminin was significantly increased from 3 to 28 days after injury, and fibronectin was continuously increased from 4 h to 28 days after injury. Compared with those of the normal mice, the levels of IL-1β and IL-6 in the *epha4*^−/−^ mice were significantly decreased, and compared with that of the control group, the expression of IL-1β and IL-6 increased significantly from 4 h to 7 days after injury. Compared with those of the normal mice, the levels of GFAP and CSPG in the *epha4*^−/−^ mice were significantly decreased, and compared with that of the control group, the expression of GFAP and CSPG increased significantly from 1 to 7 days after injury (Fig. 3a). These results indicated that EphA4 inhibits the secretion of neurotrophic factors, promotes the secretion of adhesin and cytokines, and promotes the formation of glial scars.

Primary astrocytes cultured in vitro were simultaneously used to observe the effect of EphA4 on astrocyte function. EphA4 Fc inhibited the secretion of NGF and bFGF in damaged astrocytes. The expression of NGF was significantly higher than that of the control group at 4 and 12 h post-injury, while the expression of bFGF was significantly higher than that of the control group at 12 and 24 h post-injury. Furthermore, EphA4 Fc inhibited the secretion of laminin and fibronectin in the astrocytes after injury. The expression of laminin was significantly higher than that of the control group from 12 to 72 h after injury, while the expression of fibronectin was significantly higher than that of the control group from 4 to 72 h after injury. EphA4 Fc promoted the secretion of IL-1β and IL-6 in damaged astrocytes, and the expression of IL-1β and IL-6 was significantly higher than that of the control group from 1 to 72 h after injury. Moreover, EphA4 Fc promoted the secretion of GFAP and CSPG in damaged astrocytes. The expression of GFAP was significantly higher than that of the control group from 1 to 72 h after injury, while the expression of CSPG was significantly higher than that of the control group from 4 to 72 h post-injury (Fig. 3b). These results indicated that EphA4 inhibits the secretion of neurotrophic factors and adhesin by astrocytes and promotes the release of cytokines and the formation of glial scars by astrocytes.

Since the ephrin-B family contains three ligand subtypes, we investigated the function of EphA4 binding with different ligand subtypes by interfering with ephrin-B1, ephrin-B2 and ephrin-B3. The results showed that interference with ephrin-B1 promoted the expression of NGF, bFGF, laminin and fibronectin in astrocytes and inhibited the expression of cytokines, GFAP and CSPG (Fig. 3c); interference with ephrin-B2 did not affect the secretion of neurotrophic factors, adhesion molecules, cytokines or CSPG (Fig. 3d), and interference with ephrin-B3 promoted the secretion of neurotrophic factors in astrocytes but did not affect the expression of adhesion molecules, cytokines, GFAP and CSPG (Fig. 3e and f).

### EphA4 Regulates Neurite Outgrowth and Regeneration by Binding with Ephrin-B Ligands in Astrocytes

**Fig. 4:**
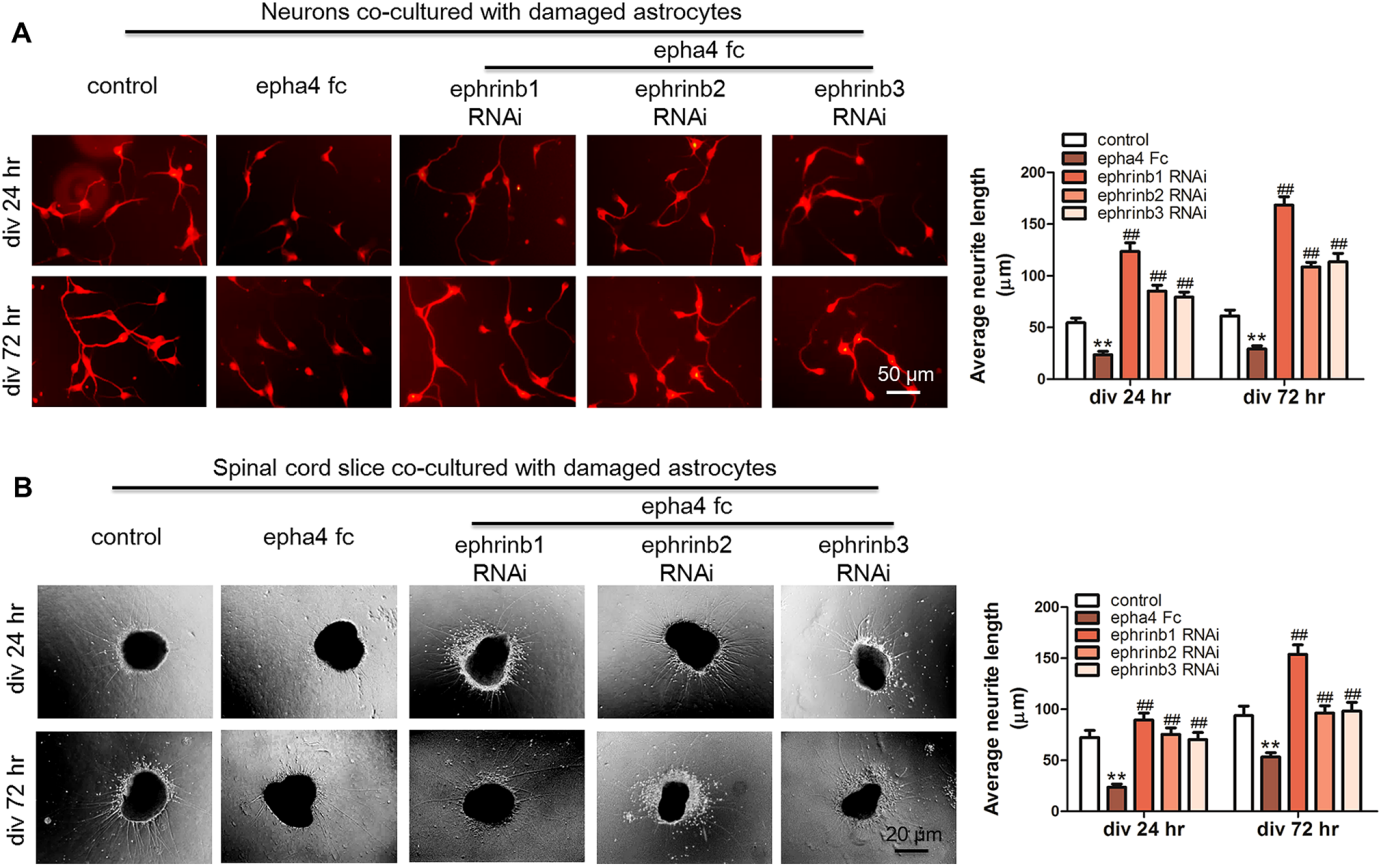
EphA4 regulates neurite outgrowth and regeneration by binding with the ephrin-B ligand of astrocytes. **a** In the neuron and damaged astrocyte coculture system, EphA4 blocked neurite outgrowth, and interference with ephrin-B ligands on the surface of astrocytes significantly promoted neurite outgrowth. **b** In the spinal cord slice and damaged astrocyte coculture system, only interference with ephrin-B1 promoted neurite regeneration in the spinal cord slices, however, interference with ephrin-B ligands on the surface of astrocytes significantly promoted neurite outgrowth with the EphA4 Fc stimulation. *N* = 5; *^, #^*P* < 0.05, **^, ##^*P* < 0.01, *vs. control, ^#^vs. epha4 Fc. Scale bar: 20 or 50 μm

Using a coculture system of neurons and astrocytes, we observed the effect of EphA4 binding with different ephrin-B ligands on neurite outgrowth and regeneration in a damaged astrocyte culture system. The results showed that interference with ephrin-B ligand on the surface of astrocytes significantly promoted neurite outgrowth (Fig. 4a), while only interference with ephrin-B1 promoted neurite regeneration in spinal cord slices in the coculture system (Fig. 4b), however, interference with ephrin-B ligands on the surface of astrocytes significantly promoted neurite outgrowth with the EphA4 Fc stimulation. These results indicated that the interaction between EphA4 and ephrin-B1 plays an important role in neurite outgrowth and regeneration.

### Effects of Inhibition of Cytokines and CSPG in Astrocytes on the Regulation of Neurite Outgrowth and Regeneration

**Fig. 5:**
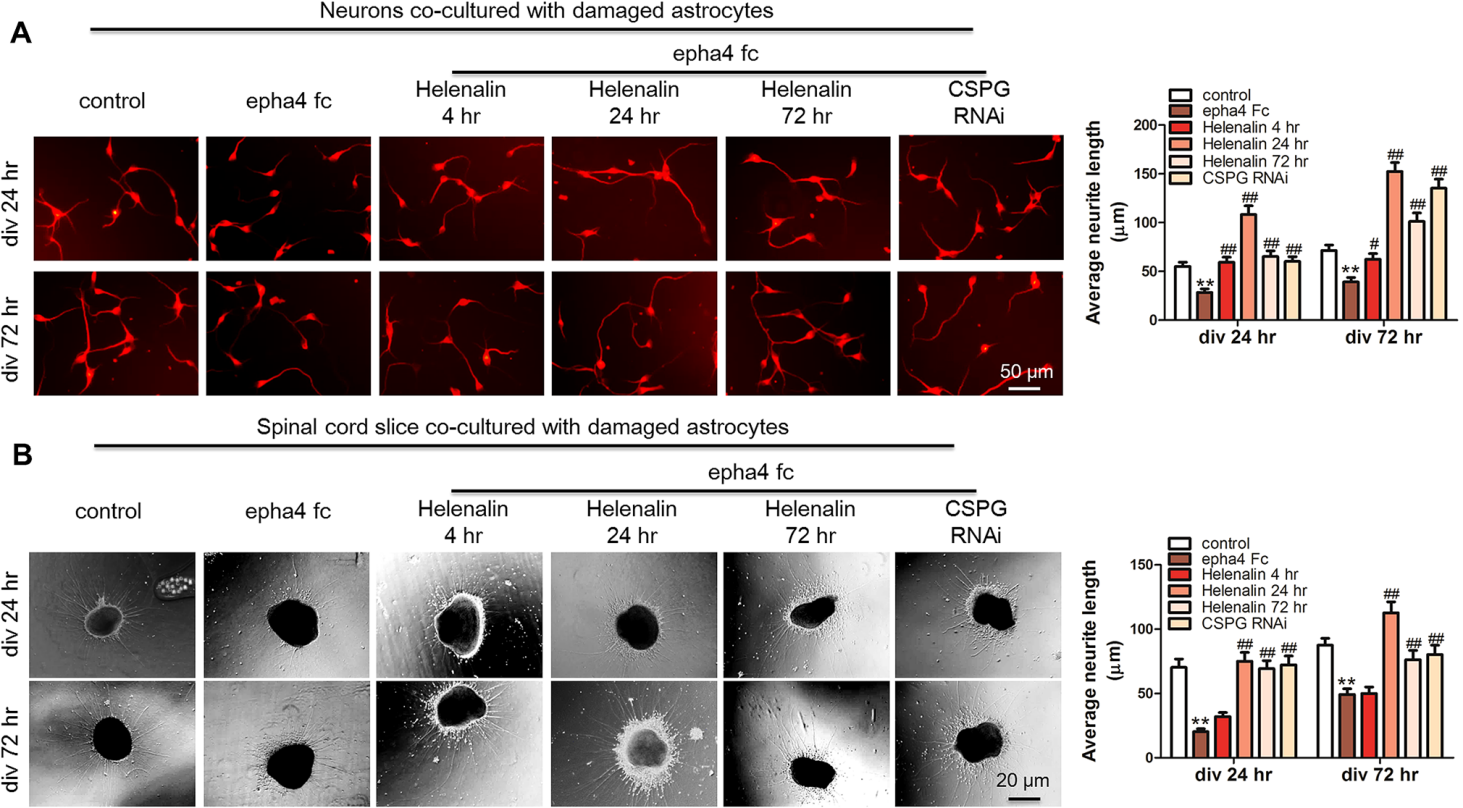
The effect of inhibition of cytokines and CSPG in astrocytes on the regulation of neurite outgrowth and regeneration. **a** In the neuron and damaged astrocyte coculture system, the interference of inflammatory factor expression at 4 h and deletion of CSPG after astrocyte injury promoted neurite outgrowth. **b** In the spinal cord slice and damaged astrocyte coculture system, the interference of inflammatory factor expression at 24 h after astrocyte injury was only conducive to promoting neurite regeneration, however, interference with inflammatory factor and CSPG expression significantly promoted neurite outgrowth with the EphA4 Fc stimulation. *N* = 5; *^, #^*P* < 0.05, **^, ##^*P* < 0.01, *vs. control, ^#^vs. epha4 Fc. Scale bar: 20 or 50 μm

We examined the effects of interfering with cytokines on neurite outgrowth and regeneration in the coculture system at different time points, and the results showed that interfering with the secretion of cytokines at 24 h and 72 h after astrocyte injury could promote neurite outgrowth but not neurite regeneration. Interference with the cytokine expression at 4 h after astrocyte injury did not promote neurite regeneration (Fig. 5a). Interfering with the expression of CSPG alone only promoted neurite outgrowth but not neurite regeneration (Fig. 5b). However, interference with inflammatory factor and CSPG expression significantly promoted neurite outgrowth with the EphA4 Fc stimulation.

## Discussion

Neuronal apoptosis and necrosis, astrocyte proliferation and glial scar formation, microglial migration and necrotic cell clearance, oligodendrocyte demyelination and axonal conduction disorder are all involved in functional recovery after SCI. Moreover, glial cells provide a favourable environment for the survival and regeneration of neurons and limit damage to these processes (Jain et al. 2015; Ishikawa et al. 2014). As the most widely distributed astrocytes in the CNS, astrocytes have the important physiological function of nutritional protection and regulation of neuronal function and are involved in excessive proliferation and formation of glial scars after CNS injury (Gaudet and Fonken 2018). The excessive proliferation of astrocytes after injury is believed to be a mechanism to protect neurons and address damage (limiting the scope of damage). However, excessive protection hinders the regeneration of neuronal processes and functional reconstruction. Strategies to use this double-edged sword reasonably to seek a balance between promoting neurite regeneration and limiting the damage will be critical for the functional recovery after SCI. Eph receptors and their ligand family members (ephrins) are the largest receptor tyrosine protein kinase subfamily and have an important function in the nervous system of embryos and adults. After SCI, Eph family members and their ligands (ephrins) accumulate in the proximal axon stump, and reactive astrocytes play an important role in the formation of glial scars and neurite regeneration (Elgebaly 2020). EphA4 reduces the expression of glutamate transporters and the glutamate absorption capacity of astrocytes by activating the reverse signal transduction of ephrin-A3, which indicates that EphA4-mediated ephrin-A3 reverse signal transduction is an important mechanism by which astrocytes control glial glutamate transporters and prevent glutamate excitotoxicity under pathological conditions (Baudet et al. 2020). In *epha4*^−/−^ mice, the number of astrocytes and the area of glial scars were significantly reduced after lateral spinal cord hemisection (Dixon et al. 2012; Fiore et al. 2019), and neurons cocultured with wild-type astrocytes exhibited shorter neurites than those cocultured with *epha4*^−/−^ astrocytes (Goldshmit et al. 2011). In contrast, some studies reported that *epha4* gene deletion did not significantly affect the reactive proliferation of astrocytes or promote the formation of astrocyte fibrosis scars after spinal cord hemisection in mice (Dixon et al. 2012); therefore, how EphA4 regulates astrocyte function and how EphA4 affects neurite regeneration and functional recovery after SCI need to be further studied.

Studies have shown that EphA4 activation leads to the retraction of dendritic spines and reduces their number and size. Ephrin-A3 on the surface of astrocytes is particularly important for EphA4 activation. In transgenic mice overexpressing ephrin-A3, especially in astrocytes, the EphA4 tyrosine phosphorylation level was significantly increased, which enhanced the effect of EphA4 in inhibiting axon growth and regeneration (Carmona et al. 2009). At present, most studies have indicated that EphA4 plays a role in inhibiting neurite outgrowth and regeneration. EphA4 receptor activation induced the retraction of dendritic spines and reduces the density of dendritic spines (Fu et al. 2014), and the upregulation of EphA4 expression in the injured axons resulted in axonal retraction and inhibited axonal regeneration several weeks after SCI (Goldshmit et al. 2004). After SCI, EphA4 was highly expressed in the axons of the corticospinal tract, ephrin-B2 was expressed in astrocytes around the corticospinal tract, and ephrin-B2 inhibited the regeneration of injured axons through the reverse signalling pathway. The axons of the *epha4*^−/−^ mice were less responsive to the growth inhibition signal of ephrin-B2 than those of the control mice, and the number of regenerated axons was significantly increased; moreover, the promotional role of axon regeneration of ephrin-B3 was partly offset by the inhibition of EphA4 (Fabes et al. 2006). Because EphA4 binds to all ephrin-B ligands and is involved in a complex two-way regulatory mechanism, our research focused on elucidating the function and the regulatory role of EphA4 in astrocytes, which will contribute to our understanding of the function of EphA4 after SCI and elucidate how astrocytes participate in repair post-injury.

Astrocytes, as the largest group of cells in the CNS, have many physiological functions, such as regulating the blood–brain barrier, promoting glutamate uptake, supporting peripheral neurons and promoting neuronal synaptic formation (Alvarez et al. 2013; Halassa and Haydon 2010). Astrocytes have many processes that stretch and fill the space between neuronal bodies and processes to support and separate neurons; furthermore, the end of astrocyte processes expands to form the terminal foot, which adheres to the adjacent capillary wall, participates in the formation of the blood–brain barrier, and absorbs nutrients from the blood to supply neurons (Bayraktar et al. 2014). After SCI, to respond to changes in the local microenvironment and generate defensive responses to injury, astrocytes undergo severe morphological and gene expression changes and become reactive astrocytes. Reactive astrocytes and other cell debris participate in the formation of glial scars (Su et al. 2011). Previous studies suggested that reactive astrocyte hypertrophy and aggregation in glial scars seriously hindered the regeneration and functional reconstruction of injured neuronal processes (Bradbury et al. 2002; Silver and Miller 2004; Rhodes and Fawcett 2004). Do glial scarring and inflammatory reactions play multiple roles after SCI (Rolls et al. 2009; Kawano et al. 2012)? Anderson et al*.* found that astrocyte scarring promotes axonal regeneration after SCI (Anderson et al. 2016), and controversial research showed that moderate reactive proliferation of astrocytes is beneficial to neurite regeneration after injury. However, how to control the reactive proliferation of astrocytes in the range conducive to neurite regeneration remains to be further studied. Some studies have shown that astrocytes at different distances from the centre of SCI have different reactivities; those closer to the centre had greater morphological changes and more secreted inhibitory factors, and the reactive astrocytes far away from the injury centre eventually return to the resting state with a normal morphology and function (Malhotra et al. 1993). The pathological process of SCI can be divided into the acute stage (0–2 days post-injury), subacute stage (2–14 days post-injury) and chronic stage (2–24 weeks post-injury). In the acute stage, activated and proliferated astrocytes migrate to the damaged area to participate in tissue repair and the inflammatory response; in the subacute stage, glial scar formation, excessive inflammatory reactions, and axon demyelination are not conducive to damage repair; and in the chronic stage, necrosis and glial scarring also hinder the regeneration of neurites (Zamanian et al. 2012; Gallo and Deneen 2014). Therefore, it is very important to select the appropriate time point to intervene according to the functional state of astrocytes in different stages after SCI and to regulate reactive astrocytes to the state conducive to neurite outgrowth. We found that EphA4 combined with different ephrin-B had different regulatory effects on astrocytes, and the effects of inhibiting inflammatory reactions on neurite outgrowth and regeneration were different at different time points. Therefore, we believe that it is very important for functional recovery after SCI to select the appropriate time point for intervention according to the functional status of astrocytes at different stages after SCI and to regulate reactive astrocytes to a state conducive to neuronal damage and repair. Our research found that alteration of astrocyte function at different times after injury had different effects on neurite outgrowth and regeneration, and the absence of ephrin-B1 is most beneficial to neurite outgrowth and regeneration.
